# Long-term disease stability with bicalutamide in a man with aggressive angiomyxoma: case report and state of art

**DOI:** 10.3389/fonc.2023.1260668

**Published:** 2024-01-16

**Authors:** Andrea Franza, Eleonora Gusmaroli, Chiara Fabbroni, Raffaella Vigorito, Sandro Pasquali, Paolo Giovanni Casali, Roberta Giovanna Sanfilippo

**Affiliations:** ^1^ Department of Medical Oncology, Fondazione IRCCS Istituto Nazionale dei Tumori, Milan, Italy; ^2^ Department of Radiology, Fondazione IRCCS Istituto Nazionale dei Tumori, Milan, Italy; ^3^ Molecular Pharmacology Unit, Department of Applied Research and Technological Development, Fondazione IRCCS Istituto Nazionale dei Tumori, Milan, Italy; ^4^ Sarcoma Service, Department of Surgery, Fondazione IRCCS Istituto Nazionale dei Tumori, Milan, Italy; ^5^ Department of Oncology and Hematology-Oncology, University of Milan, Milan, Italy

**Keywords:** angiomyxoma, neoplasm (MeSH term), connective and soft tissue neoplasms, aggressive angiomyxoma (AA), myxoma, therapy

## Abstract

Aggressive angiomyxoma (AA) is a rare mesenchymal neoplasm, which is commonly diagnosed in females and located in the perineal and pelvic region. Tissue specimens of AA patients often show positivity for estrogen (ER) and progesterone receptors (PgR), while some cases of androgen receptor (AR) positivity have been reported in males. When feasible, surgical excision represent the most effective treatment of AA; however, when experiencing advanced or recurrent disease, local disease control could be achieved with systemic hormonal treatment. To date, evidence regarding AA management in male patients is scarce, and only a few cases have been reported in literature. Hereby, we describe the case of a 59-year-old-man suffering from perineal AA with positivity for androgen receptors (AR) showing a long-lasting disease stability during the treatment with an AR-blocking drug (bicalutamide). A literature review regarding the state of art of AA management with a particular look to male patients is also provided.

## Introduction

Aggressive angiomyxoma (AA) is a rare mesenchymal neoplasm, commonly arising from the perineum and pelvic region. AA was firstly described as a separate entity among soft tissue tumors in 1983 by Steeper and Rosai, which chose the term “aggressive” to emphasize its propensity to local invasion and the high rates of recurrence (1). To date, several hundred other cases have been reported in the literature, showing that AA commonly affects females in their reproductive age. AA was even more rarely observed in men, originating from the scrotum, inguinal region and perineum (2–4).

AA appears like a grossly, deep-seated, gelatinous mass. Histologically, it consists of spindle or stellate cells scattered into a wide myxoid stroma with a predominant vascular component; mitotic count is usually low, while neoplastic cells show immunoreactivity for desmin, vimentin, smooth muscle and muscle-specific actin, and also for estrogen receptors (ER) and progesterone receptors (PgR) (1, 5, 6). HMGA2 chromosomal translocations are possible (7). Differential diagnosis includes other soft tissue tumors with secondary myxoid changes, like angiomyofibroblastoma, myxoid smooth muscle tumors, lipomatous tumors, peripheral nerve sheath tumors, myxofibrosarcoma, and pelvic fibromatosis (1). Distant metastases have been exceptionally observed (8).

Surgery is the mainstay of AA treatment but, in almost half of cases, local recurrence will occur, even after complete resections. For locally advanced/recurrent disease, systemic hormonal therapy has been investigated as an option to achieve local disease control) (9).

Considering male patients, data regarding hormonal receptor status and pharmacological activity of hormonal therapy are anecdotical (9–11).

Hereby, we report the case of a male patient with AA of the perineum obtaining a sustained radiological stability to second-line anti-androgenous therapy with bicalutamide after fast progression on letrozole.

## Case presentation

In September 2018, a 59-year-old-man with persistent low back pain, refractory to non-steroidal anti-inflammatory drugs (NSAIDs), was seen at our unit. A CT scan was performed, with the evidence of a large perineal mass, which was promptly biopsied. The histological examination revealed the diagnosis of perineal aggressive angiomyxoma. The hormonal status was positive for estrogen, progesterone and for androgen receptors.

At first, given the exclusive local aggressiveness of the disease and the location of the mass, possibly requiring mutilating surgery, in agreement with the patient we opted for a watchful waiting approach.

**Figure 1:**
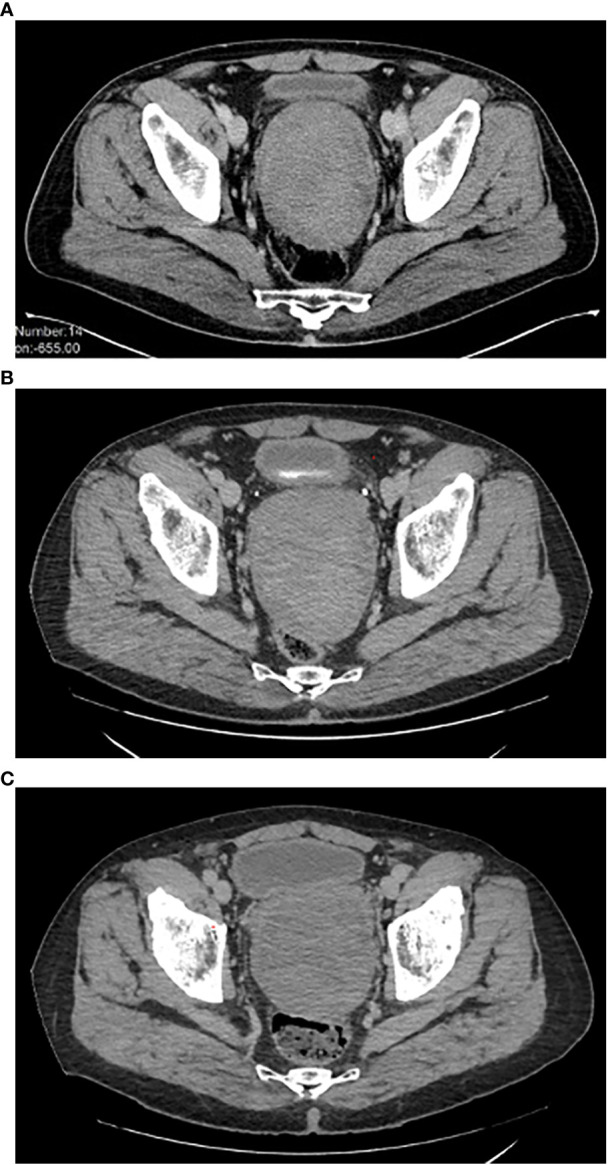
**(A-C)** Disease imaging (CT-scan). **(A)** Disease status at diagnosis. **(B)** Disease status at the time of bicalutamide start. **(C)** Disease status after 32 months of bicalutamide therapy. CT, computed tomography.

The initial size of the perineal lesion was 10x9x13 cm (**Figure 1A**), with a disease stability lasting about 12 months during watchful waiting. However, in December 2019, the mass began to increase in size (12x10x14 cm).

Therefore, a systemic treatment with an aromatase inhibitor (letrozole, 30 mg daily p.o.) was started. After three months of treatment (March 2020), the patient underwent a new CT scan, which documented further disease progression (**Figure 1B**). Low back pain also worsened, with an increasing need for painkiller administration.

The case was discussed in a multidisciplinary setting: considering the risk and the future implications of a mutilating surgery, another medical attempt was made. Given the positive receptor status, a double androgenic blockade was proposed, but patient refused due to foreseeable side effects. He was then put on bicalutamide alone at the dose of 150 mg daily p.o. in April 2020, with a prompt substantial reduction of low back pain and a significant quality-of-life improvement. Periodic (every 3-4 months) imaging assessments with abdominal and thoracic CT scan were performed, documenting a substantial radiological stability, confirmed at the last follow-up of December 2022, 32 months since the start of bicalutamide therapy (**Figure 1C**).

Treatment is still ongoing, being well tolerated. No significant adverse effect was observed, and therapy has been regularly administered daily. The patient is not taking any painkiller drug anymore and laboratory exams (blood cell values, hepato-renal function, electrolytic balance, inflammatory status) are normal. When interviewed about his quality of life, the patient reported a substantial improvement in symptoms since the start of treatment with bicalutamide and no significative limitations in his daily routine activities, working and personal relationships.

**Figure 2:**
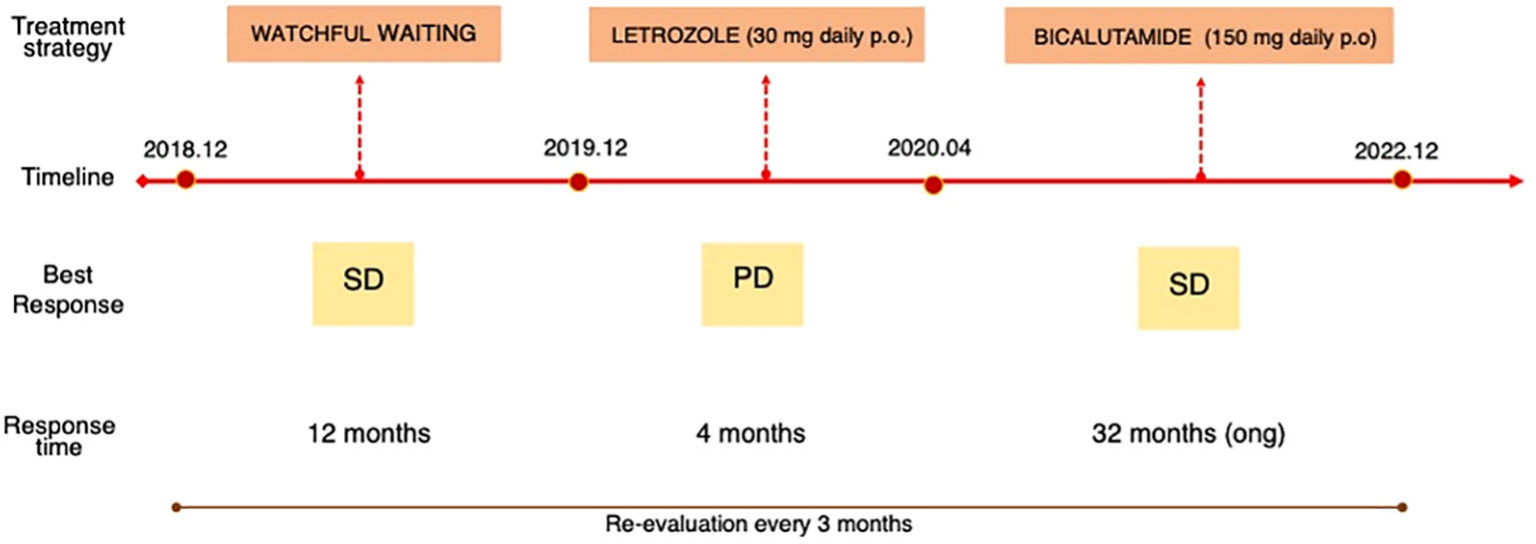
Timeline *p.o, per* os; *SD*, stable disease; *PD*, progressive disease; *ong*, ongoing.

Due to persistent disease stability, the instrumental follow-up is now scheduled every 5-6 months (**Figure 2**).

## Literature review

Historically, the mainstay of AA management was represented by surgery, with a remarkably high rate of recurrence (up to 50% of cases) (1, 9, 12). Given that the vast majority of AA exhibit positive stains for estrogen and/or progesterone receptors, systemic therapy with anti-estrogen agents has been established as the first-line approach in almost all recurrent or unresectable patients (13). Due to the extreme rarity of this condition and the small number of patients treated, only empirical evidence of pharmacological disease sensitivity is available (13, 14).

On these bases, in 2018 we conducted a multicenter, international, retrospective study in patients with AA treated with hormone therapy for locally advanced disease. In this work, most pts (36 patients included, 13 received first-line hormone therapy) were treated with a GnRH agonist (GnRHa), showing an overall response rate (ORR) of 62% and a median progression-free-survival (mPFS) of 24 months. Two complete responses (CRs), six partial responses (PRs) and five stable diseases (SDs) were registered, with the opportunity to perform a surgical resection in 1 out of 4 patients due to tumor shrinkage. The administration of an aromatase inhibitor (AI) in addition to GnRHa resulted in a new tumor response in 2 patients who progressed at fist line therapy. However, nearly all patients included in this analysis (28 out of 36) were females. The 6 male patients showed a lower prevalence of estrogen and progesterone receptor positivity. Only one male patient received first-line hormone therapy, obtaining SD as his best response and showing a PFS of 11 months (9).

Evidence regarding treatment of males with AA is scarce, with only a few reports available in literature (2, 10, 11, 15). Androgen receptor (AR) positivity has been observed in male patients, but to our knowledge no anti-androgen therapy was reported (16, 17).

The pathogenic role of androgens and their implications in therapeutical management have been broadly investigated in prostate cancer: in this neoplasm, the most classical first-line approach is represented by the combination of an anti-androgenic drug together with pharmacological castration (i.e. using an LHRH analogue) (18, 19). Over time, many compounds have been tested and there are now seven FDA-approved anti-androgens (i.e. Apalutamide, Bicalutamide, Darolutamide, Enzalutamide, Flutamide, Nilutamide and Abiraterone acetate) (20). Therapy with anti-androgenics is usually well tolerated: typical adverse effects are related to androgen - deprivation activity (i.e. flushes, erectile disfunction, gynecomastia, reduced libido) (21–23). Resistance mechanisms to androgen axis blockade, and new possible pharmacological targets, are currently under investigation (24). Several aberrations in AR signaling have been described (i.e. AR over-expression, amplifications, mutations, increased expression of androgen-synthesizing enzymes) in this setting (24). Of note, PI3K/Akt/mTOR pathway has been investigated in the setting of advanced prostate cancer progressing to androgen-deprivating therapy (ADT), with alterations found in nearly 100% of cases (25). A cross-talk between PI3K and androgen signaling pathway has been postulated, suggesting the possible role of combining mTOR inhibitors together with anti-androgenous drugs to enhance signaling inhibition of both ways (25–27).

There has been a growing interest for the administration of AR-blocking drugs in several neoplasms in the last few years. In addition to prostate cancer, evidence has been provided for salivary duct carcinoma with positivity of AR receptors and, more recently, for desmoplastic small round cell tumor (DSRCT) (28, 29). Pre-clinical models have shown improved response rate and so-called synthetic lethality of ADT combined with other pharmacological agents, such as ae (PARP) inhibitors in prostate cancer and BRAF/MEK inhibitors in melanoma (30, 31). A phase III clinical trial comparing talazoparib plus enzalutamide versus placebo plus enzalutamide in mCRPC patients (TALAPRO-2) is currently ongoing (32).

## Discussion

In this case, we observed a prolonged response (>30 months) to androgen-deprivation therapy in a male patient with perineal aggressive angiomyxoma rapidly progressing to anti-estrogen agents and not amenable to surgery. To our knowledge, this is the first report of bicalutamide administration in the disease.

In contrast to other rare malignancies, AA has been related with remarkably high overall survival. This is due to its inherent tendency to local relapse without metastasizing and directly causing death of patients (1). On the other side, local recurrence is highly associated with quality of life worsening and difficult pain control (3, 9). Previous surgery and toxicities of medical treatment could also influence and possibly exacerbate symptoms.

In this setting, when experiencing progressive disease in AA patients, clinicians should discuss which could be the best treatment option, balancing pharmacological toxicities and possible future limitations due to surgical excision, with the objective to obtain a life-long disease control.

In our patient, systemic treatment with an anti-estrogen agent (i.e. letrozole) did not show any kind of disease control, while, as soon as bicalutamide therapy was started, a clinical benefit with an arrest of progression was achieved: this observation would suggest an androgenic driver for this tumor. Interestingly, the administration of bicalutamide alone has apparently obtained a sufficient and long-term de-activation of androgen signaling, with an-over 30 months lasting response. This response duration is quite similar to the one observed by Fucà and colleagues in female patients, and remarkably high if compared with male patients in the same study (9).

In addition, our patient did not experience any bicalutamide-related adverse effect during the whole treatment course.

## Conclusion

As we pointed out before, the main objective in AA patients is to obtain a long-term disease control to avoid detrimental effects on quality of life and pain control: considering all this, our observation could be relevant for clinicians, representing the very first evidence of a possible role for AR-blocking agents in AA male patients.

Obviously, this is just a case report, in a disease, however, on which scarce data are available in literature and only a few reports take into account male patients. It should be added that now second-generation anti-androgens (i.e. apalutamide, darolutamide, enzalutamide and abiraterone acetate) are the standard of care in prostate cancer management (33). We opted for bicalutamide at the time we started treating this patient due to a lack of access to these drugs. It is also to be recalled that our patient refused LH-RH analogues. It is clear that evidence from other androgen-dependent neoplasms, like prostate cancer, could help conceive future medical approaches to this rare, challenging and so peculiar disease, when exceptionally occurring in males.

## Data availability statement

The raw data supporting the conclusions of this article will be made available by the authors, without undue reservation.

## Ethics statement

Written informed consent was obtained from the individual(s) for the publication of any potentially identifiable images or data included in this article.

## Author contributions

AF: Conceptualization, Methodology, Writing – original draft, Writing – review & editing. EG: Writing – review & editing. CF: Writing – review & editing. RV: Writing – review & editing. SP: Writing – review & editing. PC: Conceptualization, Supervision, Writing – review & editing. RS: Conceptualization, Supervision, Writing – review & editing.
